# A Microscopic Shell Structure with Schwarz’s *D*-Surface

**DOI:** 10.1038/s41598-017-13618-3

**Published:** 2017-10-17

**Authors:** Seung Chul Han, Jeong Myung Choi, Gang Liu, Kiju Kang

**Affiliations:** School of Mechanical Engineering, Chonnam National University, Gwangju, Republic of Korea

## Abstract

Recently, materials with micro-architecture of hollow trusses and ultralow density (less than 10^–2^ Mg/m^3^) have gained attention. The materials have been fabricated by forming templates based on 3D lithographical techniques, followed by hard coating on the surface, and finally etching out the template. Here, we describe a novel fabrication method for another micro architecture composed of a single continuous smooth-curved shell with *D*-surface, named Shellular; its template is prepared based on weaving flexible polymer wires. Compression test results reveal that these *D*-surfaced Shellulars have strength and Young’s modulus comparable to those of their hollow truss-based competitors. The best virtue of this weaving-based technology is its mass-productivity and large-size potential. Also, this technology can be applied to fabricate not only *D*-surfaced but also *P*- or *G*-surfaced Shellular. The unique geometry of Shellular, composed of a single continuous, smooth, and interfacial shell or membrane separating two equivalent sub-volumes intertwined with each other, appears to possess huge application potential such as non-clogging tissue engineering scaffolds and compact light-weight fuel cells with high energy density.

## Introduction

**Figure 1:**
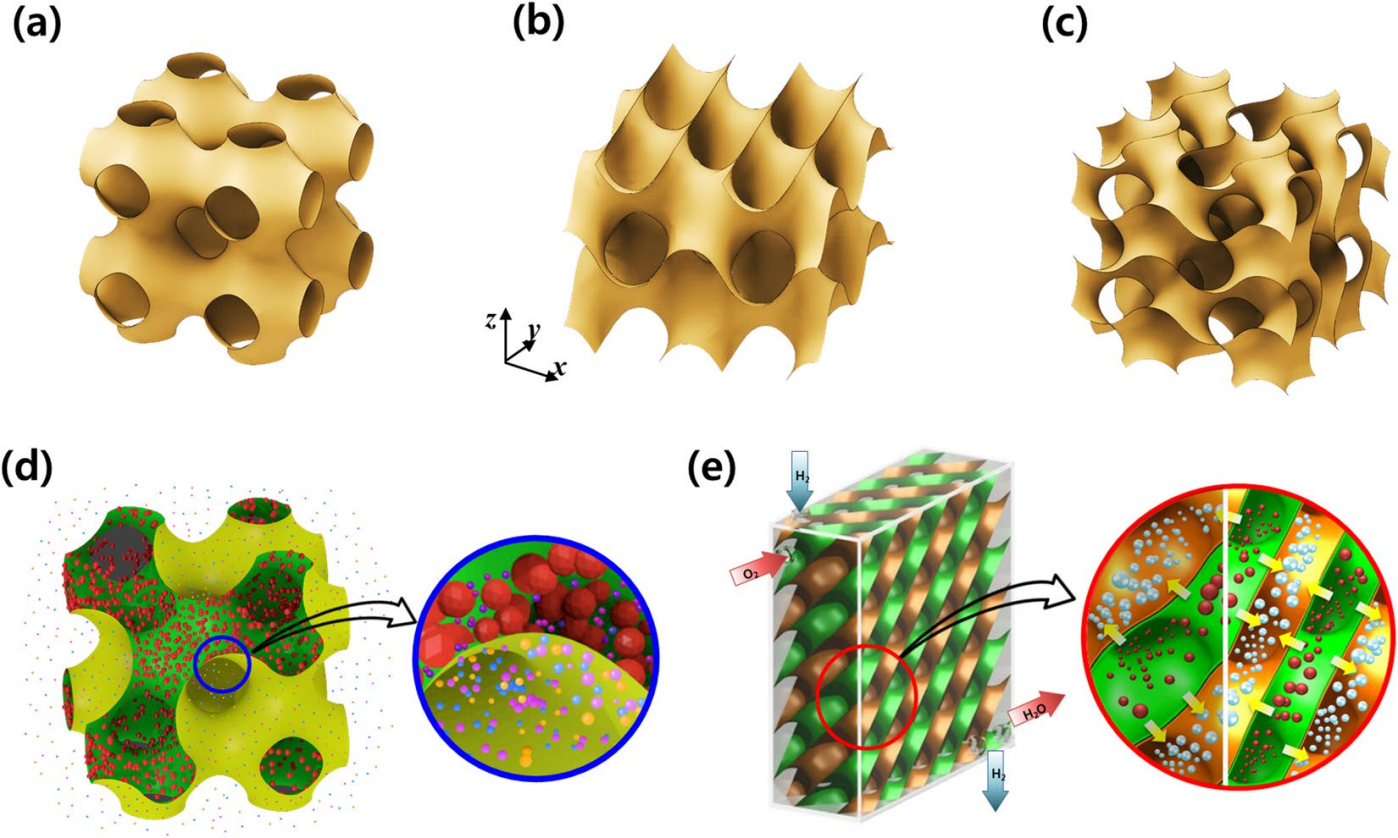
Shellular: a new type of cellular material composed of a single, smooth, continuous thin shell. Three possible options for the configurations with triply-periodic minimal surfaces (TPMS): (**a**) *P* (Primitive), (**b**) *D* (Diamond), (**c**) *G* (Gyroid)-surfaces. Two examples of potential applications: (**d**) a semipermeable membrane for osmosis in scaffolds for tissue engineering and (**e**) an interfacial shell of proton exchange membrane in a fuel cell.

Hermann Schwarz first described a Triply Periodic Minimal Surface (TPMS) in 1865^1^. A TPMS has zero (or “constant” in a wider sense) mean curvature everywhere over the entire surface, while being periodic in three directions. Also, TPMS has no self-intersections, and it partitions space into two disjoint but intertwined sub-volumes that are simultaneously continuous. Figure 1(a) to (c) depict the configurations of three typical examples of TPMS, well-known for their simplicity: *P* (Primitive), *D* (Diamond) and *G* (Gyroid)-surfaces. In the real world, examples of TPMS include some biological structures in nature, block copolymers and electrostatic equipotential surfaces in crystals^1^. Actually, most TPMS forms exist as an interface between two phases.

Since the initial introduction of Microlattice in 2011^2^, followed by Nanolattice^3,4^, and Mechanical metamaterial^5^, this new class of materials having “micro-architecture”, defined by Fleck *et al*.^6^, as well as ultralow density (less than 10^−2^ g/cc) has become a hot research issue. Fabrication of these materials involves forming polymeric templates, coating hard materials on the surface of the templates, and subsequent etching out of the templates to leave hollow truss architectures. These materials have much higher strength and stiffness than previous cellular materials with stochastic structures at a given relative density, because of their periodic hollow truss architectures comprised of strong constituent materials (i.e., metallic or ceramic films with nano-sized grains). In fact, the concept of truss (or hollow truss) cellular materials was introduced in early 2000^7^, as a novel measure to increase strength and stiffness of cellular materials by inducing the ‘stretching-dominated’ deformation (i.e., axial tension or compression is acting along the straight struts composing the trussed cellular materials under external loading)^7,8^. Hence, these three materials (of Microlattice, Nanolattice, and Mechanical metamaterial) have been regarded as the most advanced versions of trussed cellular materials. These three materials differ only in the UV lithographic technology used to form the polymeric templates.

Most recently, another micro-architectured and ultralow density material, named ‘Shellular’ was introduced^9^. Instead of the hollow trusses, Shellular is composed of a thin continuous shell. We believe that TPMS might represent a good choice as the architecture of Shellular. In other words, because a TPMS has a smooth surface with constant mean curvature, a thin shell of TPMS does not have stress concentration due to geometrical irregularity, but rather can support an external load by coplanar stresses, without causing bending. Hence, the thin shell in a TPMS can serve as another stretching-dominated structure^10^, in addition to the trussed cellular materials, generally referred to as micro-architectured materials^6^.

In addition, we expect a thin continuous shell of Shellular in a TPMS to play a role as a transfer interface between the two sub-volumes, as well as a mechanical load support. The interfacial shell can be a semipermeable membrane for osmosis in scaffolds for tissue engineering, or an electrolyte membrane for fuel cells or rechargeable batteries. Figure 1(d) and (e) depict the conceptual models of these scaffold and fuel cell that are composed of semipermeable membrane and proton exchange membranes, respectively. These two examples will be elaborated in “Discussion” section. Thus, these porous systems with two void sub-volumes intertwined throughout each other, and separated by the thin interfacial shell with a smooth and large surface appear to have huge potential. However, according to our best knowledge, no one had ever fabricated or even suggested such a novel architecture, prior to Han *et al*.^9^ introducing the concept of Shellular in a TPMS configuration.

The fabrication of the Shellular specimens by Han *et al*.^9^ followed that of Microlattice in terms of template-forming by a 3D UV lithographic technology, the self-propagating polymer waveguide^11^. The compression tests on the Shellulars revealed that their strengths and stiffness held up as comparable to their three predecessors (i.e., Microlattice and Nanolattice, and Mechanical metamaterials). However, the lithographical technique could produce a template with an overall thickness less than approximately 10 mm, and it could be used to fabricate a Shellular with only *P*-surface among a TPMS family. Even intended to be *P*-surfaced, in reality the morphology was substantially elongated in the vertical direction, due to a limitation of the lithography using an out-of-plane illumination of UV through a mask.

## Concept of Fabrication Method

**Figure 2:**
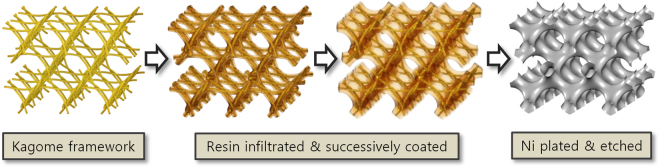
A schematic of the fabrication process of *D*-surfaced Shellular based on a wire-woven Kagome structure.

In this paper, to overcome the limits of the lithographical methods, mentioned above, we describe a novel method to form a template for Shellular in a TPMS configuration. Unlike the previous micro-architectured and ultralow density materials (with templates formed by lithographical techniques), we prepared the template for this new Shellular by weaving a framework using flexible polymer wires, filling the wire framework with resin, then subsequently curing the resin. The rest of the process remains identical to that in the predecessors mentioned above. Figure 2 shows a schematic of the fabrication process. The wire framework used for this Shellular could be chosen among a family of wire-woven structures^12^ developed by our research group in the last decade. We chose a Kagome truss-like structure to fabricate Shellulars with *D*-surface (shown in Fig. 1(b)), having the largest surface area and highest mean curvature among the three representative morphologies of TPMS shown in Fig. 1(a) to (c) ^13^. We expect the large surface area to provide an enhanced potential for applications as the transfer interfaces, mentioned above. By seeking the optimal shape and the proper processes (for resin-filling, post-treatment, coating, and etching), we fabricated a new micro-architectured and ultralow density material, Shellular with a *D-*surface; we also evaluated its mechanical properties by compression tests, making comparisons with its predecessors.

*D*-surface is defined by the following equation^14^;

1 $$\begin{array}{c}\sin (\frac{2\pi x}{a})\times \,\sin (\frac{2\pi y}{a})\times \,\sin (\frac{2\pi z}{a})+\\ \sin (\frac{2\pi x}{a})\times \,\cos (\frac{2\pi y}{a})\times \,\cos (\frac{2\pi z}{a})+\\ \cos (\frac{2\pi x}{a})\times \,\sin (\frac{2\pi y}{a})\times \,\cos (\frac{2\pi z}{a})+\\ \cos (\frac{2\pi x}{a})\times \,\cos (\frac{2\pi y}{a})\times \,\sin (\frac{2\pi z}{a})+k=0\end{array}$$

with the coordinates, *x*, *y* and *z* axes depicted in Fig. 1(b). In the equation, *a* is the cell size, and *k* is a constant adjusted for a desired value of volume fraction, *f*, defined as the ratio of the inner sub-volume to the overall volume. (The specific relation between *f* and *k* is given in the Supplementary Information). In order to determine an optimal value of *f* for the highest strength, we carried out finite element analyses on CAD models of *D*-surfaced Shellular with four different volume fractions, *f*, and two different shell thicknesses, *t*. From the results, we found the volume fraction of *f* = 0.2 to give the highest strength per relative density regardless of the failure mode. See the Supplementary Information for the technical details of the FEA analysis and the results. Henceforth, we prepared the specimens with a value of volume fraction as close as possible to *f* = 0.2.

## Results

**Figure 3:**
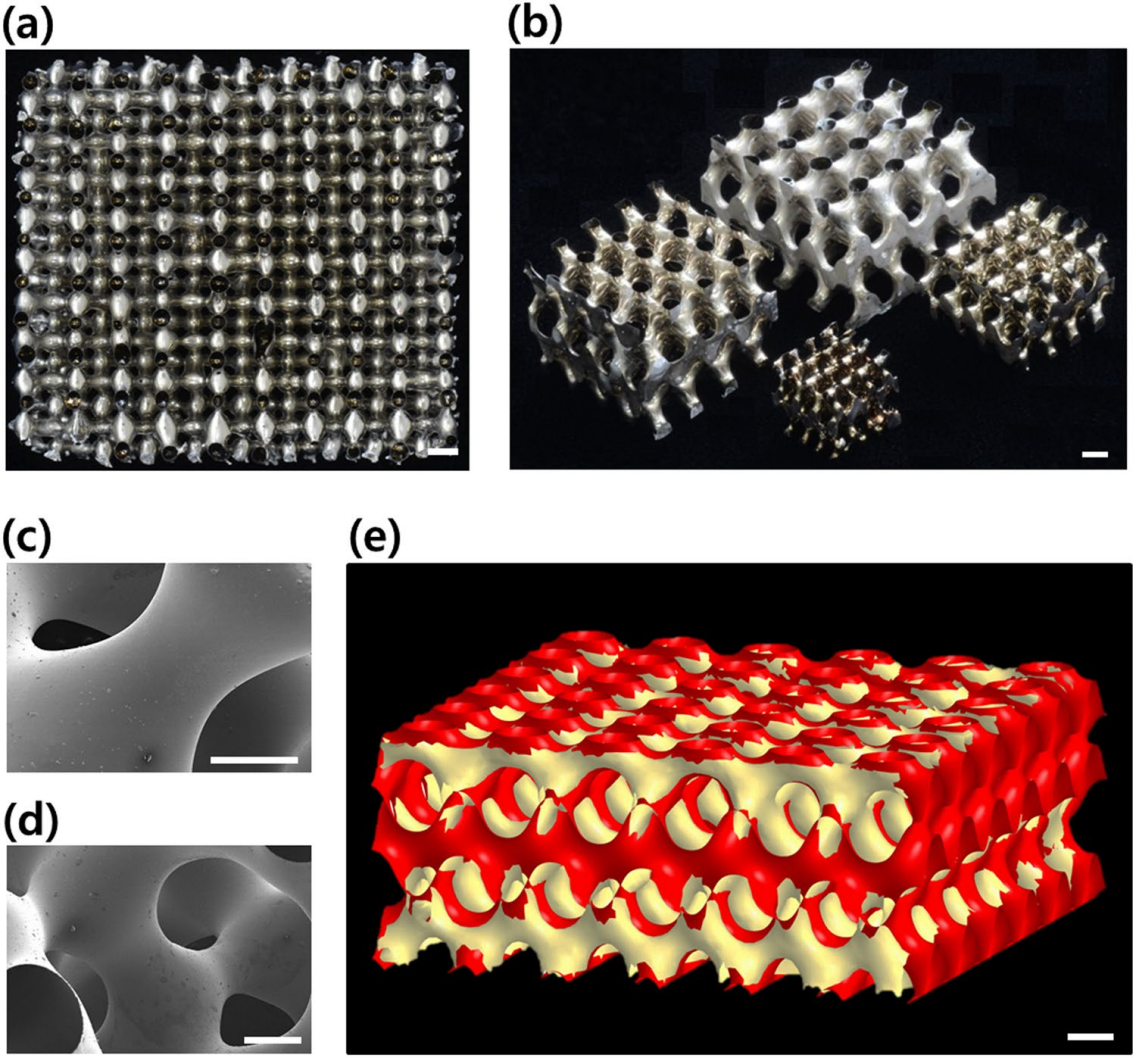
Final architecture of the *D*-surfaced Shellular specimens. (**a**) and (**b**) Optical images: (**a**) top view; (**b**) isotropic views for four different cell sizes. (**c**) and (**d**) SEM images revealing the smooth surfaces of the Ni-P shells. (**e**) Micro-CT image overlapped with a CAD image of the *D*-surface with a volume fraction of *f* = 0.25, according to Equation (1). Scale bars, 3 mm (**a**,**b**), 800 $\mu {m}$ (**c**,**d**), 2 ${mm}$ (**e**).

Figure 3(a) and (b) show examples of the completed specimens. Figure 3(c) and (d) show SEM images revealing their decidedly smooth surface. A 3D image (i.e., micro-CT image) was obtained using an X-ray micro-tomography (Quantum GX, Perkin Elmer, Inc., USA). Figure 3(e) shows the micro-CT image (yellow) overlapped with a CAD image (red) of the *D*-surface with a volume fraction of *f* = 0.25 according to Equation (1), demonstrating that the configuration of a specimen was fairly well-matched to the theory (i.e., revealing good agreement between the two images). A movie illustrating the 3D configuration of the specimen measured by the micro-CT is provided in Movie S2 of Supplementary Information.

**Figure 4:**
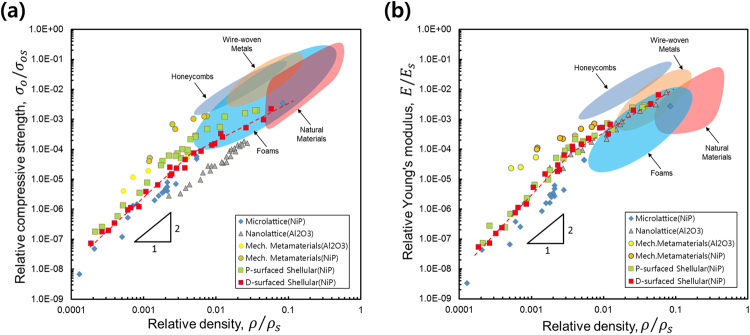
Measured material properties of the *D*-surfaced Shellulars in comparison with three competitors and conventional cellular materials. (**a**) Relative compressive strengths; and (**b**) relative Young**’**s moduli according to relative densities.

The detailed results of the mechanical properties measured from the compression tests for the *D*-surfaced Shellular specimens are given in Figure S6 and Movies S3 and S4 with a subsection of the Supplementary Information. Figure 4(a) and (b) show the relative compressive strengths and relative Young’s moduli, respectively, according to the relative densities. For comparison, the properties of the three competitors, namely, Microlattice^2^, Nanolattice^3,4^, and Mechanical metamaterial^5^, are shown together. In the figures, the property domains of the conventional cellular materials: engineering honeycombs^15^, wire-woven metals^12^, natural materials^16^, and foams^16^ are also indicated. These figures show that the lower bound of relative densities of the conventional cellular materials is around $\,(\tfrac{\rho }{{\rho }_{s}})\sim 0.01$.

The strengths and Young’s moduli of the *D*-surfaced Shellulars were higher than or comparable to those of the Microlattice or Nanolattice; however, both measured properties were still substantially lower than those of the Mechanical Metamaterials. We attributed the lower strengths and moduli observed in the experiments to the geometrical incompleteness and material defects of the specimens. Composed of a single continuous smooth thin shell, Shellular’s mechanical properties easily deteriorate due to local incompleteness or defects. In contrast, being composed of hollow trusses, its counterparts (e.g., Microlattice, Nanolattice, and Mechanical Metamaterials) have intrinsic barriers in their geometries against incompleteness or defects (i.e., when a defect exists in a hollow truss element, the influence will likely be blocked by the abruptly changing geometry at nearby joints between the truss elements). Especially, the individual unit cells of Nanolattice and Mechanical Metamaterials are rigid (i.e., statically and kinematically determinate in the architectures), and thus still able to support load, even if a few unit cells are missing^17^. Notably, among the three typical TPMSs, *D*-surface possesses the highest mean curvature, likely to induce unwanted bending or buckling early under an external load. Nevertheless, the fact that *D*-surfaced Shellulars have strength and Young’s modulus comparable to those of their hollow truss-based competitors indicates the superior concept of Shellular.

## Discussion

### Why weaving?

Weaving, used for more than 9000 years, represents one of the oldest human technologies^18^. During the industrial revolution around the 18th Century, mechanized processes rapidly advanced. Since the Second World War, various 3D weaving technologies have been developed for applications in fiber-reinforced composites. In fact, textiles can be regarded as a type of periodic cellular material. Hence, textiles or wire-woven structures can be produced with a wide range of sizes for overall and unit cell dimension and wire thickness. Presently, so-called ultra microfibers (thickness of the order of 1 μm) have attained widespread use in weaving various textiles. Hence, should ultra-microfibers be used to fabricate the Kagome frameworks, *D*-surfaced Shellular composed of unit cells of 10 μm order would become feasible.

The best virtue of this weaving-based technology is its mass-productivity and large-size potential. In fact, we have developed the weaving mechanism and succeeded in making a prototype loom. Figure S7 of the Supplementary Information shows the proto-type weaving machine and a sample woven by this machine^19^. Recently, they have proven that not only *D*-surfaced but also *P*- or *G*-surfaced Shellular can be fabricated based on a similar approach herein described^20^.

### Why not 3D printing?

One might consider additive manufacturing to form the template for TPMS Shellular, as had been done for the conventional scaffolds mentioned above. In fact, Nanolattices and Mechanical metamaterials have been fabricated based on templates formed by certain kinds of additive manufacturing. However, besides their slow formation and high cost, additive manufacturing techniques have a fatal problem with the degree of precision to be used to form the template for a TPMS Shellular, composed of a smooth and thin shell. The typical ratio of shell thickness to cell size of Shellular is about 10^−3^, as shown in the paper by Han *et al*.^9^, or in Table S1 of the Supplementary Information. In order to obtain Shellular with 10 mm of overall size and 1 mm of cell size, the wall thickness should be thinner than 1 μm. Here, again to achieve a *sufficiently smooth* surface, the precision of the additive manufacturing technique should be lower than 1/10 of the wall thickness, that is, 100 nm. Namely, the resolution in overall length scale (1 dimension) should be finer than 1/10^5^, which is definitely beyond the precision of additive manufacturing techniques. The Nanolattice^3,4^ and Mechanical Meta-material^5^, mentioned above, were fabricated using templates formed by top-notch additive manufacturing technologies (i.e., two-photon lithography-based direct laser writing^21^ and projection micro-stereo-lithography^22^), respectively. Their resolutions were 10 nm/50 μm = 1/5,000 and 5 μm/1.5 mm = 1/300, respectively, being much rougher than the required resolution of 10^−5^. Consequently, only tiny sizes of the specimens were prepared. The *sufficiently smooth* surface, having hardly been any issue in conventional 3D models, is essential in Shellular composed of a continuous thin shell, because a rough shell surface would be likely to buckle early under compressive coplanar stress.

### Potential Applications

In this work, the experimental results have revealed that *D*-surfaced Shellular has mechanical properties comparable to those of its predecessors that are composed of hollow tubes. As the first trial, metallic shell structures were fabricated through electroless plating. Shellular comprised of ceramic, polymer, and other constituent materials could be fabricated through ALD (atomic layer deposition), PVD (physical vapor deposition), CVD (chemical vapor deposition), or even dip coating, as has been done with Microlattices^23^. As mentioned above, the unique geometry of Shellular (composed of a single continuous, smooth, and interfacial shell or membrane separating two equivalent sub-volumes inter-wound with each other) appears to possess huge application potential. For example, as depicted in Fig. 1(d), *D*-surfaced Shellular could serve as a new concept for fabricating tissue engineering scaffolds composed of a biocompatible membrane made from porous natural or synthetic polymers, such as chitosan and poly L-lactic acid (PLLA)^24^. In fact, recently, porous materials with TPMS configurations have gained attention as scaffolds for tissue engineering applications. Because the TPMS possesses desirable characteristics (e.g., a smooth surface, high surface area, and excellent permeability), the scaffold in TPMS is regarded to be ideal for cell adhesion, migration, and vitalization in tissue engineering^25–27^. However, the previous scaffolds in TPMS have only one void sub-volume to serve as the place for cells to grow and simultaneously for the passage of mass (e.g., nutrients, oxygen, and waste) to be transferred, while the other solid sub-volume supports the external force. This type of scaffold appears to have gained popularity because it can be precisely fabricated by means of CAD modeling and additive engineering^28,29^. However, the cell density and thickness of the growing tissue can give rise to diffusion constraints through the void sub-volume, limiting the mass transfer to the interior of the scaffold over 200 μm from the external surface^30^.

In contrast, the Shellular scaffold has two void sub-volumes separated by a thin interfacial membrane with a continuous, smooth, and large surface. The two void sub-volumes could serve independently as the places for cells to grow and for the passage of mass to be supplied, whereas the membrane shell could play two different roles as the transfer interface of a semi-permeable material between the two void sub-volumes and as a support structure against external load. In fact, Kapfer *et al*.^27^ have shown that structures with two void sub-volumes could possess much more effective load-bearing capability than those with a single void sub-volume. Meanwhile, a similar architecture has already been proposed as a model for the corneocytes of mammalian skin with a lipid interface of *G* (Gyroid) surface, shown in Fig. 1(c), between the two void sub-volumes, with one occupied by a keratin fiber structure, while the other was filled by water^31^. We expect that this new concept of scaffold will provide a perfect passage, that never gets blocked by the growing tissue incubating in the other sub-volume separated by the interfacial porous shell.

Interfacial shells in fuel cells represent another example of the potential applications of the Shellular with *D*-surface or other TPMS. Most interfaces used for the purpose are flat membranes, being sandwiched between thick backing plates of mostly graphite. Figure 1(e) depicts a new concept for a fuel cell, with the interfacial shell comprised of electrolyte material, while its differently-colored two opposite surfaces are coated by catalysts and porous conductive layers. Air and fuel pass separately through the two void sub-volumes. Because the interfacial shell plays dual roles as both proton exchange membrane and load support, while providing a very smooth and large surface, we believe backing plates to be unnecessary; consequently, the whole system can be very compact and light-weight with high energy density. With stress concentration being minimized, we expect the smooth shell with constant curvature to soundly resist thermal expansion or contraction, as well as resist mechanical loading due to the internal gas pressure or external force.

In addition, Shellular can be used as an ultralight raw material for mechanical or civil engineering. Conventional honeycombs have intrinsically 2D structures with strength in only one direction. In contrast, Shellulars have 3D structures with near isotropic properties. Hence, we expect Shellulars to be employed as ultralight core materials of more arbitrarily-shaped bodies (rather than sandwich panels).

## Conclusions

In this study, we described a novel fabrication method for a micro-architectured material, composed of a single continuous smooth-curved shell with *D*-surface, named Shellular; its template was prepared based on weaving flexible polymer wires. By seeking the optimal shape and the proper processes (for resin-filling, post-treatment, coating, and etching), we fabricated the specimens of *D-* surfaces Shellular, and evaluated its mechanical properties by compression tests, making comparisons with its predecessors. Our conclusions are as follows:

i)   The optical, SEM, micro-CT images on the Shellular specimens revealed that the surface was quite smooth and continuous, as intended, and that the geometry was fairly well matched to the mathematical model of *D*-surface with a volume fraction of *f* = 0.25.

ii)  The *D*-surfaced Shellulars had strengths and Young’s moduli comparable to those of their hollow truss-based competitors.

iii) The best virtue of this weaving-based technology is its mass-productivity and large-size potential. Also, this technology could be applied to fabricate not only *D*-surfaced but also *P*- or *G*-surfaced Shellular.

iv) The unique geometry of Shellular, composed of a single continuous, smooth, and interfacial shell or membrane separating two equivalent sub-volumes intertwined with each other, appears to possess huge application potential such as non-clogging tissue engineering scaffolds and compact light-weight fuel cells with and high energy density.

## Method

### Specimen Preparation

#### Template Formation

Previously, we created various wire-woven structures by spin-inserting helically pre-formed metallic wires. However, for the purpose of this work, the Kagome framework should be built by literally *weaving* flexible polymer wires, so as to be easily etched out afterwards without any damage to the metallic hard coating. Hence, we used a newly-developed 3D weaving technique^15^.

Figure S8(a) shows the configuration of the tetrahedron unit cell composing a 3D Kagome framework. The unit cell consisted of four wires in the out-of-plane directions (colored yellow) and two separate wires in the in-plane directions (colored dark brown). Figure S8(b) shows a schematic of the Kagome framework woven between the transparent top plate and the bottom reed plate, excluding the external frames. Figure S8(c) and (d) show photos of the loom used to manually weave the Kagome framework, and a close-up view of the serially woven five Kagome frameworks (fixed with external frames to prevent spring-back of the wires), respectively. We used poly lactic acid (PLA) wires of 0.2 or 0.4 mm diameter to weave the Kagome frameworks with 3 and 4 mm or 5 and 6 mm pitch (the pitch became the cell size), respectively.

Figure S9 represents a series of arrangements for the magnetic buttons holding the top ends of warps through a polycarbonate top plate during the weaving process, depicted in Figure S8(b) and (c). Here, the thick dashed lines denote horizontally inserted wefts. The bottom ends of the warps were positioned by the square-mesh patterned holes on the reed plate. The warps and wefts became the struts in the out-of-plane and in-plane directions in the Kagome structure, respectively. After switching every two neighbor buttons in the x-direction on the top plate (making the couples of warps cross over each other), the wefts were inserted over the cross-points in the y-direction. Subsequently, the inserted wefts were pulled down above the reed plate. Afterward, switching the buttons in the *y*-direction, inserting the wefts in the *x*-directions, and pulling down the wefts were carried out. The same process was repeated until the uppermost wefts reached to the top plate, to obtain a multiple-layered structure. Movie S1 of Supplementary Information shows how the weaving process operated in a 2D schematic.

Thereafter, the intersections between the wires composing the Kagome framework were fixed by infiltrating a UV-curable resin, Thiol-ene. The resin was preliminary cured under UV ray, then fully hardened in an electric oven at 80 °C for 24 hours, before the external frames were removed. Figure S10(a) shows that the intersections between the wires composing the Kagome framework were fixed by infiltrating a UV-curable resin, Thiol-ene. Extra resin was applied on the Kagome framework first to fill the tetrahedron-like cells, then to smoothen the surface (similar to a *D*-surface). The parameters governing the shape of a liquid drop forming on a solid framework are the surface tension of the liquid, the intermolecular force between the liquid and the solid material, and gravity. The surface tension makes the surface area minimal, but in contrast, the intermolecular force makes the liquid adhere to the solid, resulting in a capillary phenomenon. Hence, a proper balance between these two forces should be sought, while minimizing the effect of gravity. This issue is elaborated in a subsection of the Supplementary Information with Figure S4. In this study, the smooth surface was attained by repeatedly coating and curing extra resin with low viscosity. The effect of gravity could be minimized by using only a small amount of resin for each coating, and periodically turning over the structure once a minute during curing. Finally, it was fully hardened again in an electric oven at 80 °C for 24 hours. Figure S10(b) and (c) show the top/side views and their close-ups of the completed template.

#### Ni-plating and etching

The surfaces of the template formed on the woven Kagome framework were coated with a nickel-phosphorus (Ni-P) thin film through electroless plating using a commercially available process (OM Group Inc., Cleveland, OH). After washing out the template using acetone and DI water, its surface was treated for five minutes with a solution of 0.071 mole KMnO4 and 0.167 mole NaOH at a ratio of 8:2. The surface was then coated with PT-activator for 10 min. Finally, electroless nickel plating was carried out in a bath maintained at pH 4.9 and 80 °C with Boron accelerator. The thickness of the metal coating was controlled by the plating time in a range of 1.5 min to 6 hr, yielding metal films from 300 nm to 64 μm. Thereafter, to obtain the *D*-surfaced Shellular, the interior template should be removed. First, Thiol-ene resin was etched out by a 50:50 mixture of 3 mole NaOH aqueous solution and methyl alcohol for 12 hr, and the remaining solution was then washed out by flowing DI water for 30 min. The PLA wires were etched out twice by Chloroform at 40 °C for 30 min. Finally, the remaining agents was washed out by dipping into acetone for 5 min, and then dried, leaving a clean Shellular composed of nickel foil.

### Compression tests

Figure S5(a) and (b) of the Supplementary Information show the setup for the compression tests and a specimen mounted between a pair of compression platen. The test machine was INSTRON 8872 and the displacement was controlled at a rate of 0.005 mm/s. In addition to built-in load cell, Lebow 3397-50 load cell with 200 N capacity was placed under the lower compression platen to precisely measure the force applied to the specimen. Tinius Olsen 500 L laser extensometer was used to measure the displacement. A Dino-Lite Pro AM 4223T portable microscope and a Nikon D-50 camera were used to monitor the deformation under compression.

### Data availability

The authors declare that the data supporting the findings of this study are available in Supplementary Information.

## Electronic supplementary material

Supplementary Information

Movie S1

Movie S2

Movie S3

Movie S4
