# Supplementary material for: A Microscopic Shell Structure with Schwarz’s D-Surface
Source: Sci Rep. 2017 Oct 17;7:13405. doi: 10.1038/s41598-017-13618-3 (PMC5645438; doi:10.1038/s41598-017-13618-3)
Supplement: Supplementary file 1 — Supplementary Information [file 41598_2017_13618_MOESM1_ESM.doc]

Supplementary Information for

A Microscopic Shell Structure with Schwarz’s *D*-Surface

S.C. Han, J.M. Choi, Liu Gang, *Kiju Kang

correspondence to: kjkang@chonnam.ac.kr

**This PDF file includes:**

Supplementary Text

Figures S1 to S10

Tables S1

Captions for Movies S1 to S4

**Other Supplementary Information for this manuscript includes the following:**

Movies S1 to S4

**Finite element analysis and optimal design**

The *D*-surface is defined by the following equation

$\begin{matrix} \sin\left(\frac{2\pi x}{a}\right)\times\sin\left(\frac{2\pi y}{a}\right)\times\sin\left(\frac{2\pi z}{a}\right)+ \\ \sin\left(\frac{2\pi x}{a}\right)\times\cos\left(\frac{2\pi y}{a}\right)\times\cos\left(\frac{2\pi z}{a}\right)+ \\ \cos\left(\frac{2\pi x}{a}\right)\times\sin\left(\frac{2\pi y}{a}\right)\times\cos\left(\frac{2\pi z}{a}\right)+ \\ \cos\left(\frac{2\pi x}{a}\right)\times\cos\left(\frac{2\pi y}{a}\right)\times\sin\left(\frac{2\pi z}{a}\right)+k=0 \end{matrix}$ . -- (1)

CAD simulation gives the relation between the constant, *k*, and the volume fraction, *f*, as follows;

$$k=2.691\times f-1.333$$

**Figure S1** depicts CAD models of *D*-surfaced Shellular with four different volume fractions, *f* = 0.2 to 0.5, made based on Equation (1) in the main text. Each of the models had a constant cell size, *a* = 4.444 mm, a number of 4*4*2 cells, and two different thicknesses, *t* = 1 and 10 μm. Finite element analyses were performed to simulate the mechanical behaviors subjected to compression. The thin film was modeled using 4-node general-purpose shell elements. ABAQUS v 6.12 was used as the finite element solver. For sound accuracy, the smallest possible elements were used (e.g., the size of shell elements was around 100 μm). For all of the FEA, the properties of the Shell material (Ni-P alloy) were given as follows: Young's modulus of *E_s_* = 210 GPa, Poisson's ratio of *ν_s_* = 0.3, and yield strength of *σ_os_* = 2.5GPa.

Considering the face sheets attached on the top and bottom faces to avoid local buckling (that easily occurs with the thin shell structure of Shellular), all of the nodes on both the top and bottom faces of each model were constrained from translation and rotations (except for a downward displacement on the top face); meanwhile, all of the nodes on the outermost lateral faces (i.e., on the edges of the lateral openings shown in **Figure S2**) were left free.

Eigenvalue buckling analyses with two-step linear perturbations were performed to calculate the buckling strengths of the samples. The technical details are given in the reference^9^. To calculate the yield strength of a sample, a separate elastic-plastic analysis was carried out according to *J_2_*-flow theory, taking the stress at the offset strain of *ε*_z_ = 0.002 as the yield strength. To calculate the Young’s modulus, a small displacement *δ*_z_ was applied to the face +*z* (corresponding to *ε*_z_ = -0.001%) to obtain reaction force *F_R_*. The Young’s modulus was calculated from the displacement and reaction force assuming the sample to be a homogeneous material.

**Figure S2** shows Mises stress distribution for two models with *f* =0.2 and 0.5 under a constant compressive load of 20 N, which is about a half of the first peak loads. The shell thicknesses are identical as *t* =10 μm. Both figures reveal very even stress distribution, demonstrating why a TPMS should be taken as the Shellular morphology for the high mechanical resistance.

**Figure S3** shows variations of the relative compressive strength and relative density according to the volume fraction. It was observed that, at the shell thickness of *t* = 10μm, all the models fail due to plastic yielding, whereas, at *t* = 1μm, they fail due to elastic buckling. At both shell thicknesses, as the volume fraction increased, the compressive strength decreased and the relative density, i.e., the weight increased. This means that the model of the *D*-surfaced Shellular with the lowest volume fraction of *f* = 0.2 has the highest strength and the lowest weight regardless of the failure mechanism. More detailed numerical analysis on the mechanical properties and optimal design will be carried out in near future.

**Shapes of resin infiltrated into the wire-woven structure**

**Figure S4** depicts schematic shapes of resin infiltrated into the wire-woven Kagome structures. We attributed the difference in the shapes to the relative difference between the surface tension and the intermolecular force, ignoring effect of gravity. Roughly speaking, as the surface tension increased, the shape of the resin became more like a sphere or drop, as shown in **Figure S4**(a). Contrarily, as the intermolecular force became higher, the resin more uniformly covered the wires, and the shapes became like **Figures S4**(c) and (d). A proper balance between the two forces made the resin shape move closer to the minimal surface (i.e., *D*-surface herein), as shown in **Figure S4**(b).

**Measured mechanical properties**

**Figure S6** shows stress-strain curves measured from the compression tests for four representative specimens. As observed from the previous micro architecture materials with ultralow density such as Microlattice^7^ and *P*-surfaced Shellular^14^, when the relative density was lower than a certain value, specifically *ρ/ρ_s_* ~ 10^-3^ in this case, that is, the wall thickness was much lower than the cell size, the Shellular specimen failed by elastic buckling. Otherwise, the Shellular specimen failed by plastic yielding or fracture. Consequently, **Figures S6**(a) and (b) corresponding to the specimens with the relative density lower than *ρ/ρ_s_* ~ 10^-3^ indicate no permanent deformation when fully unloaded. In contrast, **Figures S6**(c) and (d) corresponding to the specimens with the relative density higher than *ρ/ρ_s_* ~ 10^-3^ indicate some permanent deformation when fully unloaded. Because the specimens had 2 to 4 layers, most failures proceeded in layer-by-layer, which resulted in the stable behavior after the first peak in the stress-strain curves, as shown in **Figures S6**(a) to (c). **Movie S3** demonstrate the stable and fully recoverable behavior at the relative density of *ρ/ρ_s_* = 2.6* 10^-4^. However, when the relative density was higher than *ρ/ρ_s_* ~ 10^-2^, corresponding to the wall thickness higher than about 20 μm, the specimens failed by overall brittle fracture of the shell, resulting in the rapid drop after the first peak, as shown in Figures S5(d). **Movie S4** demonstrate the brittle behavior at *ρ/ρ_s_* = 1.2* 10^-2^. In near future, more detailed parametric study will be carried out on the failure mechanism and consequent mechanical properties using finite element analysis. **Table S1** lists the dimensions and properties measured for all the *D*-surfaced Shellular specimens.

**
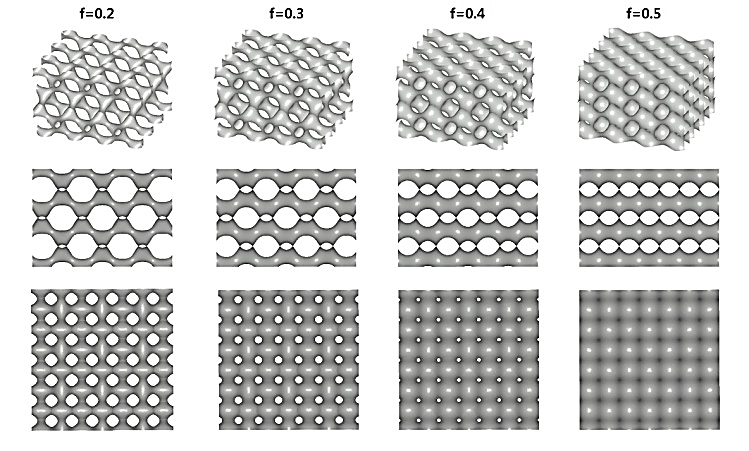
**

**Figure S1.** CAD models of *D*-surfaced Shellular with four different volume fractions.

**
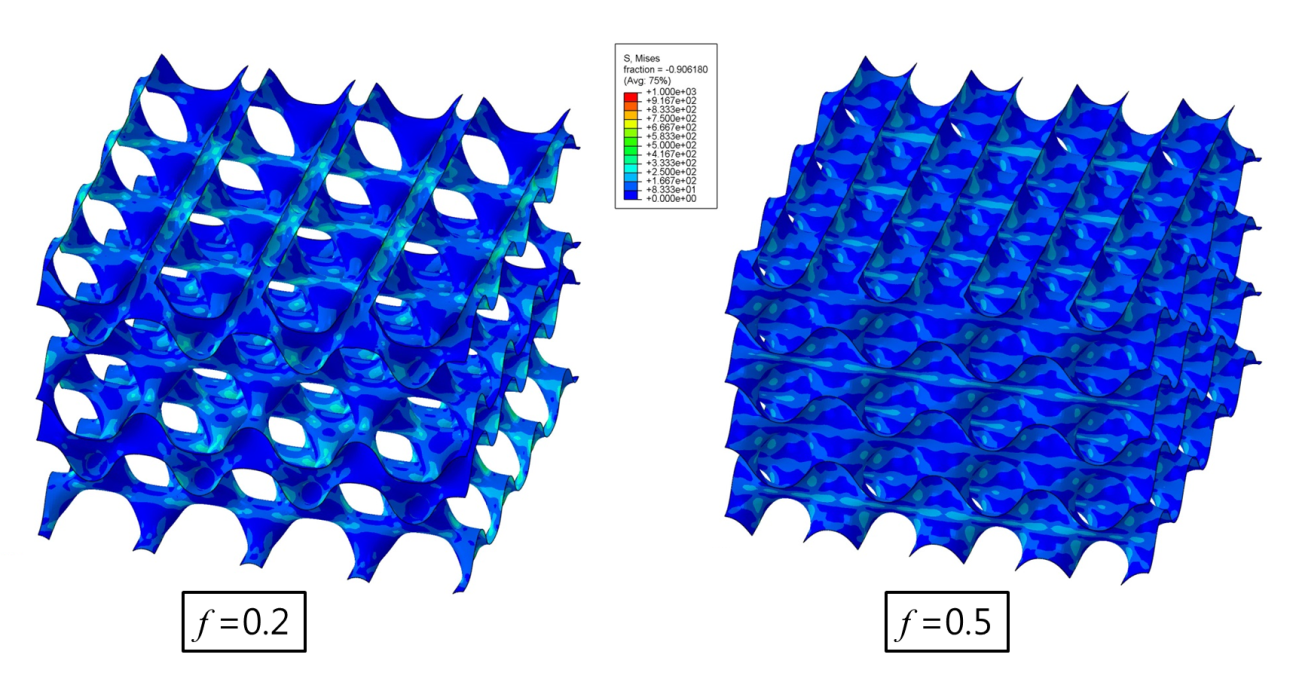
**

**Figure S2.** Stress distribution in a D-surfaced Shellular, estimated by FEA.

**
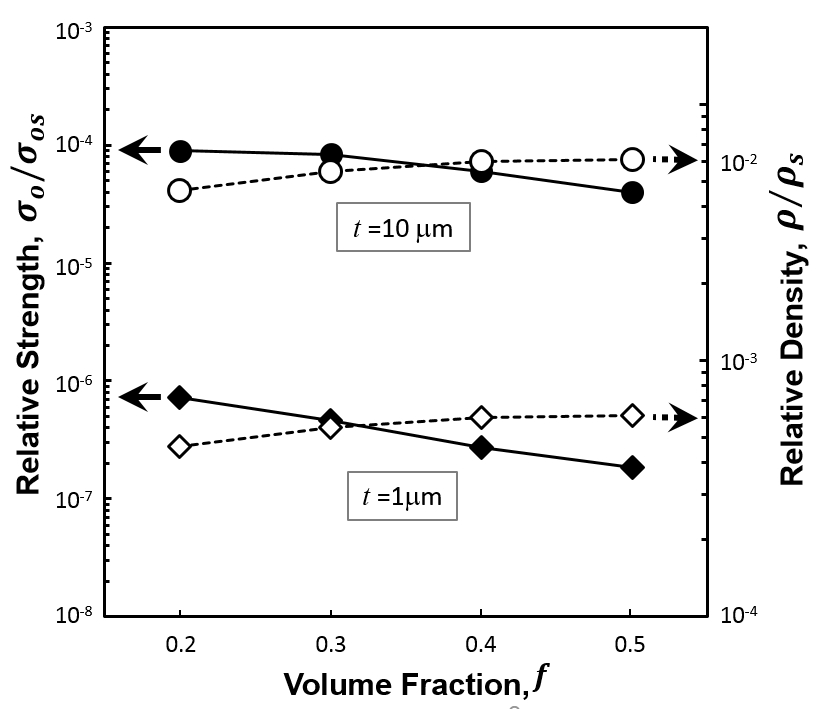
**

**Figure S3.** Variations of compressive strength and relative density according to the volume fraction at two different shell thicknesses.

**
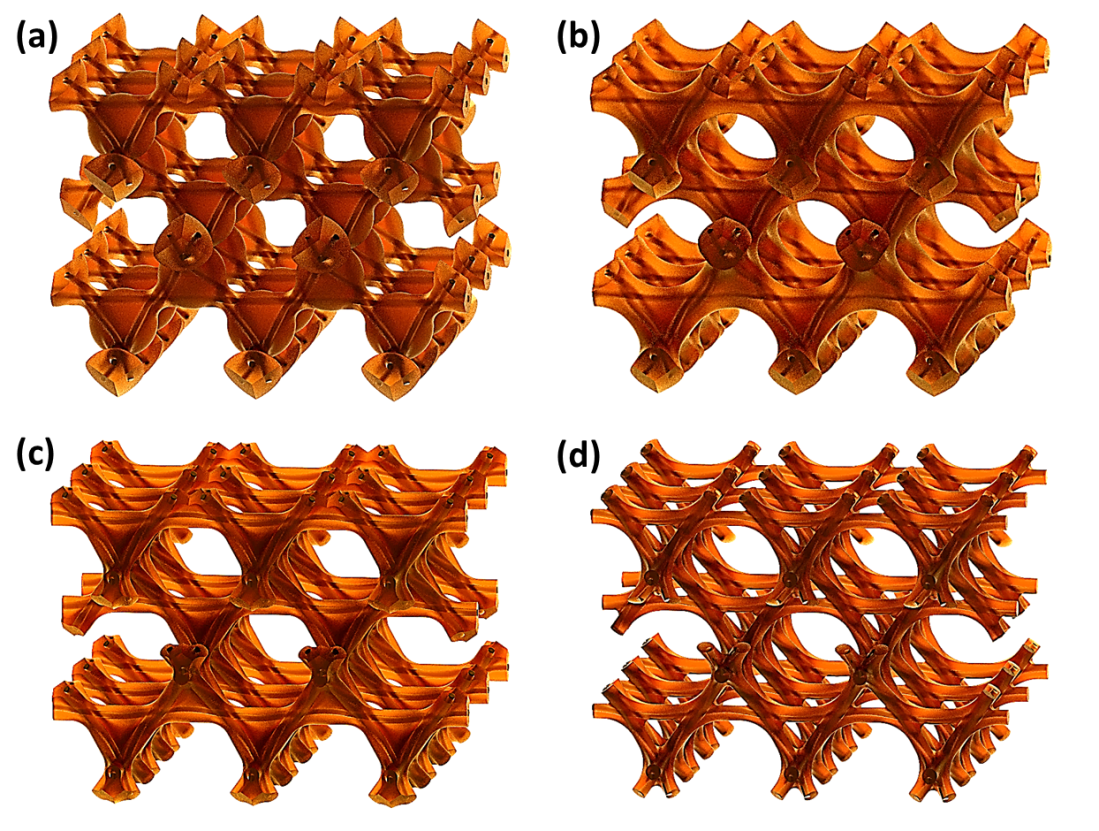
**

**Figure S4.** Variation of shapes of resin infiltrated into the wire-woven structures, depending on the two relevant forces, surface tension and intermolecular force between the resin and wires.


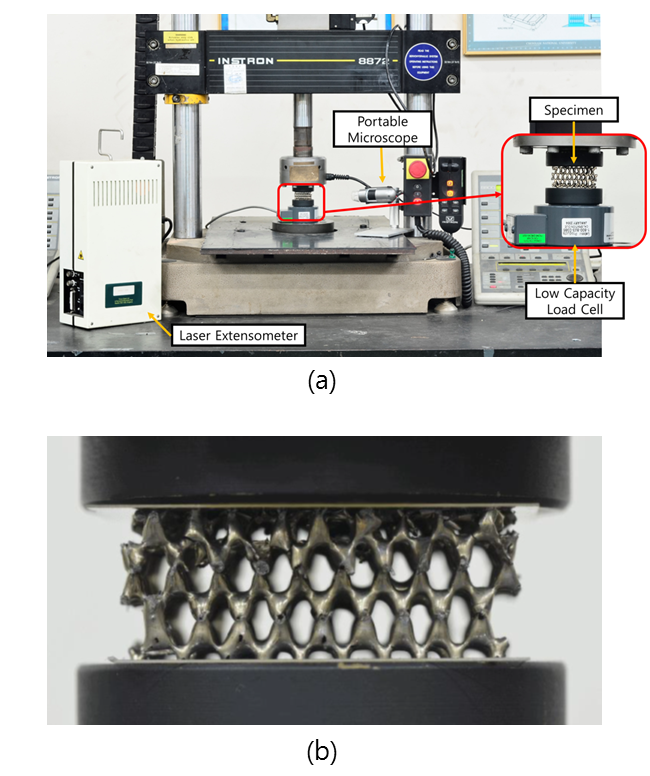


**Figure S5.** Compression tests of specimens: (a) test set-up, (b) a specimen placed between two thick platens.

**
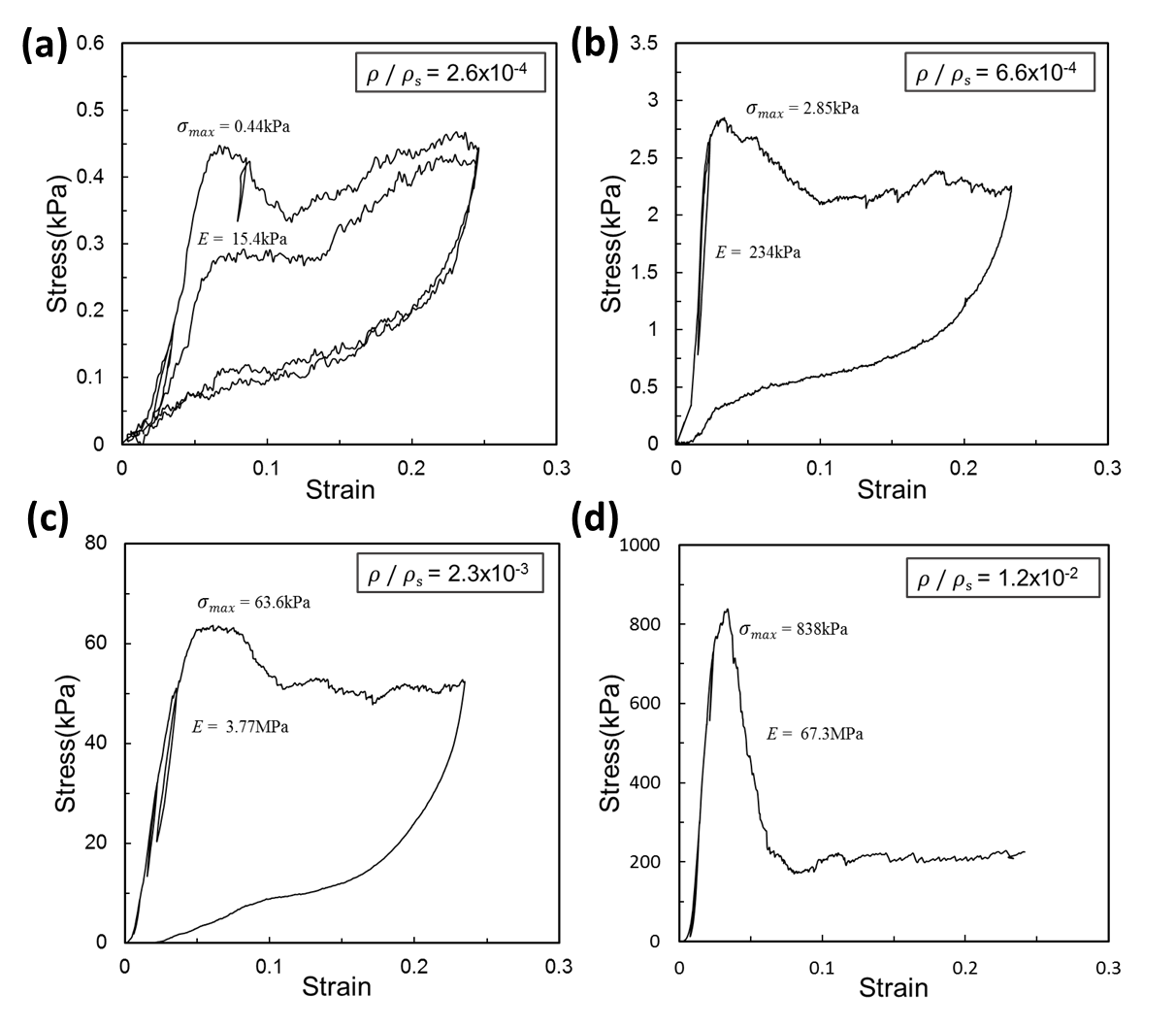
**

**Figure S6.** Stress-strain curves measured from specimens under compression tests.

**
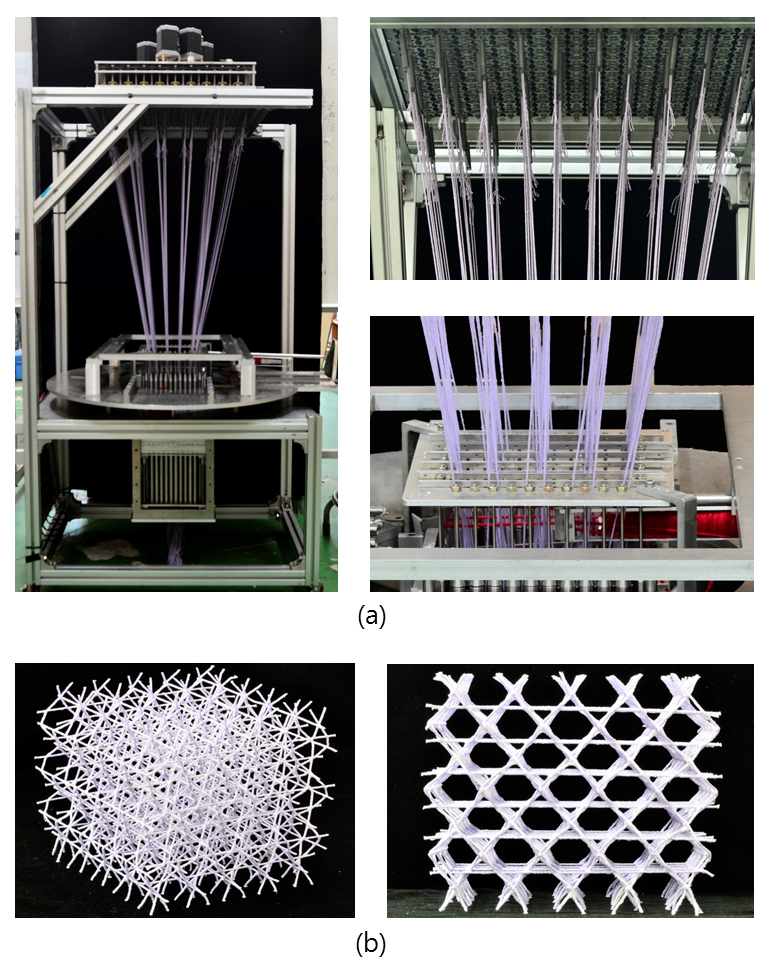
**

**Figure S7.** (a) Overall and partial views of the prototype of the weaving machine under development for mass production of Kagome textiles (b) A sample woven by this machine^15^.

**Table S1.** Dimensions and properties measured for all the *D*-surfaced Shellular specimens.

| **Wall Thickness (μm)** | **Cell Size (mm)** | **Number of Layers** | **Overall Dimensions X, Y, Z(mm)** | | | **Mass**  **(mg)** | **Density (Mg/m^3^)** | **Relative Density** | **Strength (MPa)** | **Young's Modulus (MPa)** |
| --- | --- | --- | --- | --- | --- | --- | --- | --- | --- | --- |
| 0.3 | 3 | 4 | 25.19 | 23.23 | 17.66 | 2.17E+01 | 2.10E-03 | 2.62E-04 | 4.43E-04 | 1.54E-02 |
| 1.8 | 6 | 2.5 | 27.69 | 27.2 | 17.81 | 7.03E+01 | 5.24E-03 | 6.55E-04 | 2.85E-03 | 2.34E-01 |
| 3.2 | 3 | 2.5 | 26.29 | 27.05 | 12.72 | 1.65E+02 | 1.83E-02 | 2.28E-03 | 6.36E-02 | 3.77E+00 |
| 25.4 | 5 | 3 | 22.78 | 21.81 | 21.213 | 9.84E+02 | 9.34E-02 | 1.17E-02 | 8.38E-01 | 6.73E+01 |
| 0.8 | 3 | 2 | 22.56 | 18.57 | 9.58 | 1.91E+01 | 4.77E-03 | 5.96E-04 | 2.35E-03 | 1.59E-01 |
| 0.4 | 3 | 1.5 | 22.25 | 22.3 | 6.48 | 8.29E+00 | 2.58E-03 | 3.22E-04 | 4.89E-04 | 3.22E-02 |
| 1.2 | 3 | 2.5 | 23.39 | 25.79 | 12.12 | 4.96E+01 | 6.79E-03 | 8.48E-04 | 3.08E-03 | 3.12E-01 |
| 0.2 | 3 | 2 | 22.49 | 23.86 | 9.69 | 7.67E+00 | 1.48E-03 | 1.84E-04 | 1.81E-04 | 1.13E-02 |
| 0.5 | 3 | 1.5 | 14.08 | 19.6 | 5.48 | 4.71E+00 | 3.11E-03 | 3.89E-04 | 8.70E-04 | 4.99E-02 |
| 3.4 | 4 | 3.5 | 36.16 | 36.17 | 18.78 | 4.56E+02 | 1.86E-02 | 2.32E-03 | 3.33E-02 | 6.28E+00 |
| 2.4 | 4 | 2.5 | 30.33 | 29.32 | 13.84 | 1.05E+02 | 8.53E-03 | 1.07E-03 | 9.75E-03 | 1.11E+00 |
| 5.2 | 4 | 2 | 17.33 | 16.23 | 10.47 | 6.70E+01 | 2.28E-02 | 2.84E-03 | 5.93E-02 | 1.33E+01 |
| 6.5 | 4 | 2.5 | 13.07 | 14.1 | 12.44 | 6.81E+01 | 2.97E-02 | 3.71E-03 | 6.07E-02 | 2.58E+01 |
| 16.2 | 6 | 2.5 | 21.62 | 19.34 | 19.06 | 3.96E+02 | 4.97E-02 | 6.21E-03 | 2.15E-01 | 4.81E+01 |
| 28.8 | 3 | 3 | 25.99 | 27.3 | 14.58 | 2.15E+03 | 2.07E-01 | 2.59E-02 | 1.31E+00 | 4.17E+02 |
| 5.6 | 6 | 2 | 25.33 | 20.63 | 14.02 | 1.43E+02 | 1.95E-02 | 2.44E-03 | 3.72E-02 | 4.35E+00 |
| 48.2 | 3 | 3 | 21.38 | 22.38 | 17.96 | 3.10E+03 | 3.61E-01 | 4.51E-02 | 2.40E+00 | 6.95E+02 |
| 3.4 | 5 | 2.5 | 27.5 | 25.6 | 17.67 | 1.55E+02 | 1.24E-02 | 1.55E-03 | 1.63E-02 | 3.09E+00 |
| 12.6 | 6 | 2.5 | 27.04 | 28.41 | 19.74 | 5.94E+02 | 3.92E-02 | 4.90E-03 | 1.87E-01 | 3.06E+01 |
| 8.8 | 6 | 2.5 | 27.62 | 25.34 | 19.06 | 3.99E+02 | 2.99E-02 | 3.74E-03 | 1.15E-01 | 2.04E+01 |
| 15.6 | 4 | 2.5 | 20.15 | 18.65 | 14.2 | 4.10E+02 | 7.68E-02 | 9.59E-03 | 3.80E-01 | 1.10E+02 |
| 14.2 | 4 | 2.5 | 19.25 | 17.9 | 14.22 | 3.45E+02 | 7.04E-02 | 8.80E-03 | 3.45E-01 | 1.09E+02 |
| 16.4 | 3 | 2.5 | 14.2 | 13.85 | 11.46 | 2.22E+02 | 9.83E-02 | 1.23E-02 | 6.35E-01 | 1.41E+02 |
| 62.4 | 3 | 2 | 13.93 | 14.12 | 10.28 | 9.32E+02 | 4.61E-01 | 5.76E-02 | 5.55E+00 | 1.39E+03 |


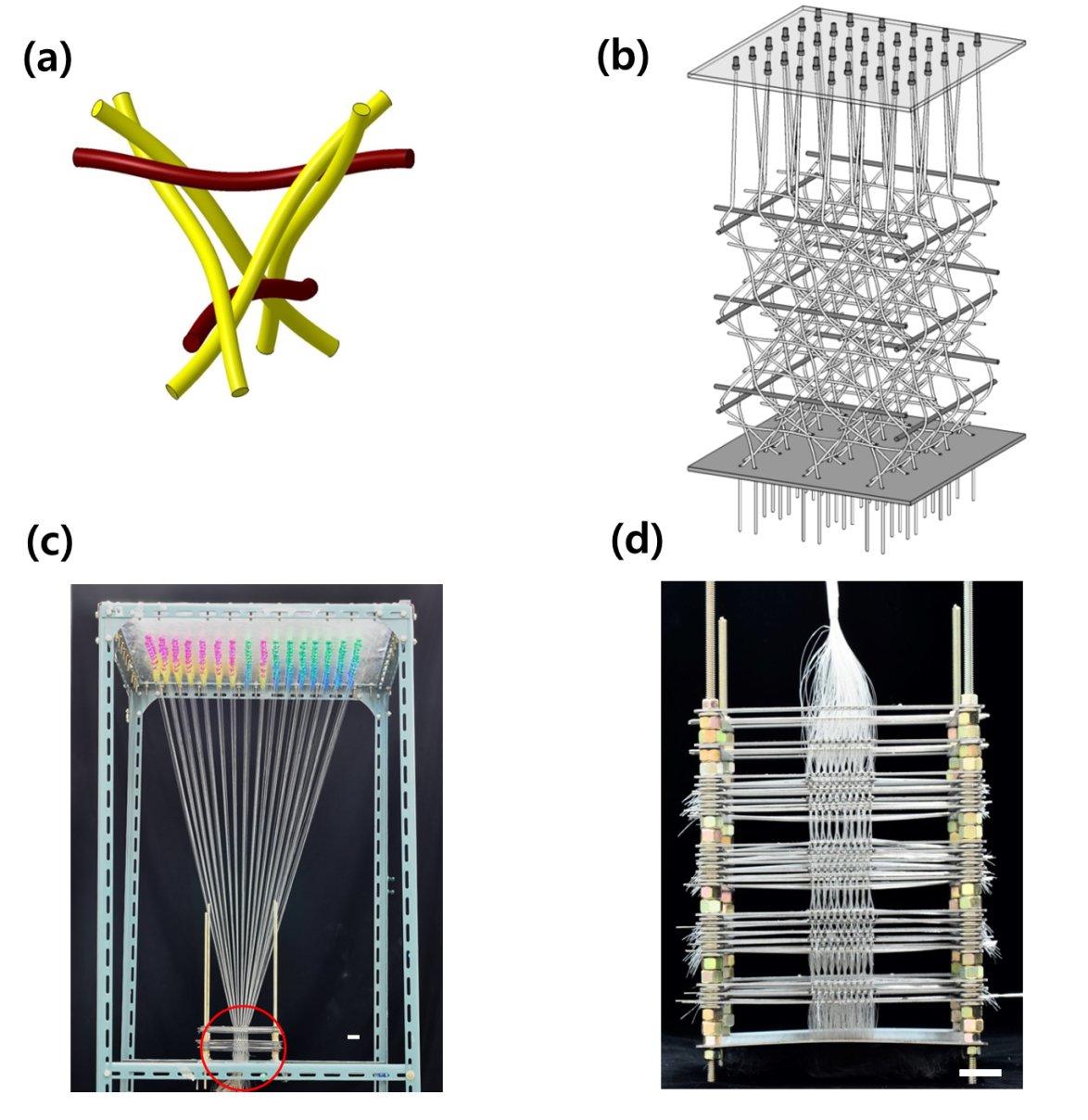


Figure S8. Kagome framework weaving. Schematics of (a) a tetrahedron unit cell composing a 3D Kagome framework and (b) the Kagome framework woven between the transparent top plate and the bottom reed plate, excluding the external frames. (c) Overall view of the loom used to weave the Kagome framework. (d) Close-up of the five serially woven Kagome frameworks, which were fixed with external frames to prevent spring-back of the wires, respectively. Scale bars, 20*mm* (a, b).


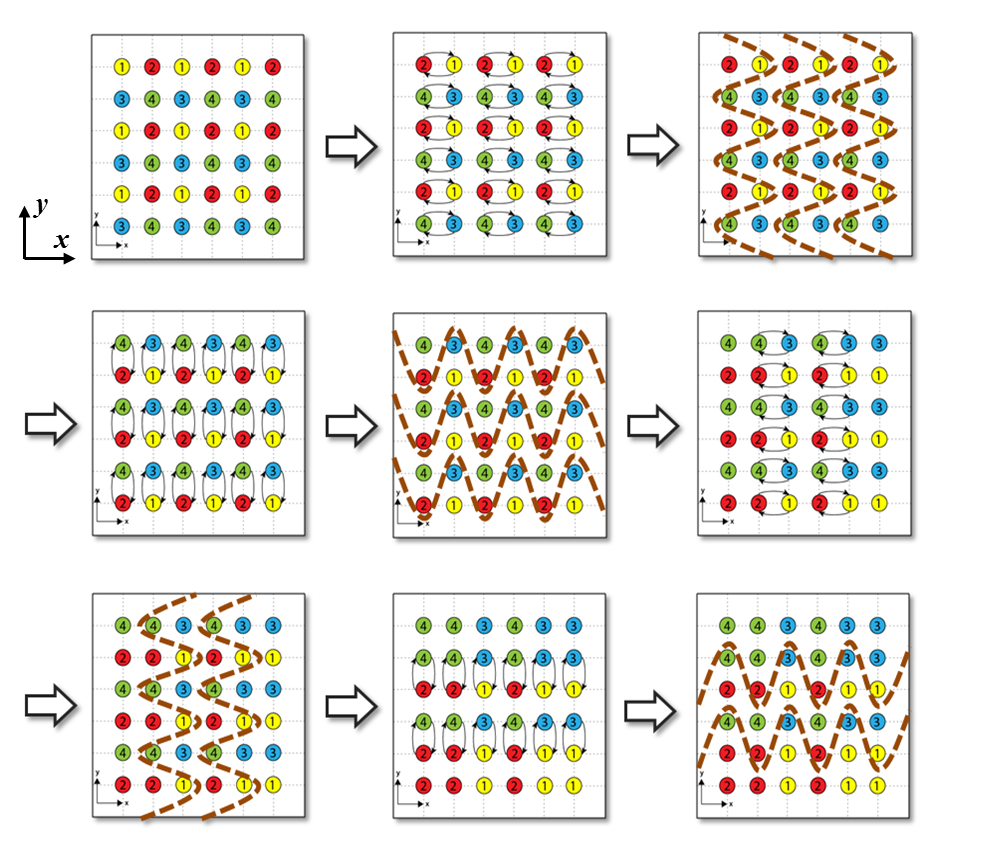


Figure S9. A series of arrangements for the magnetic buttons holding the top ends of warps through a polycarbonate top plate during the weaving process, depicted in Figures 3(b) and (c).


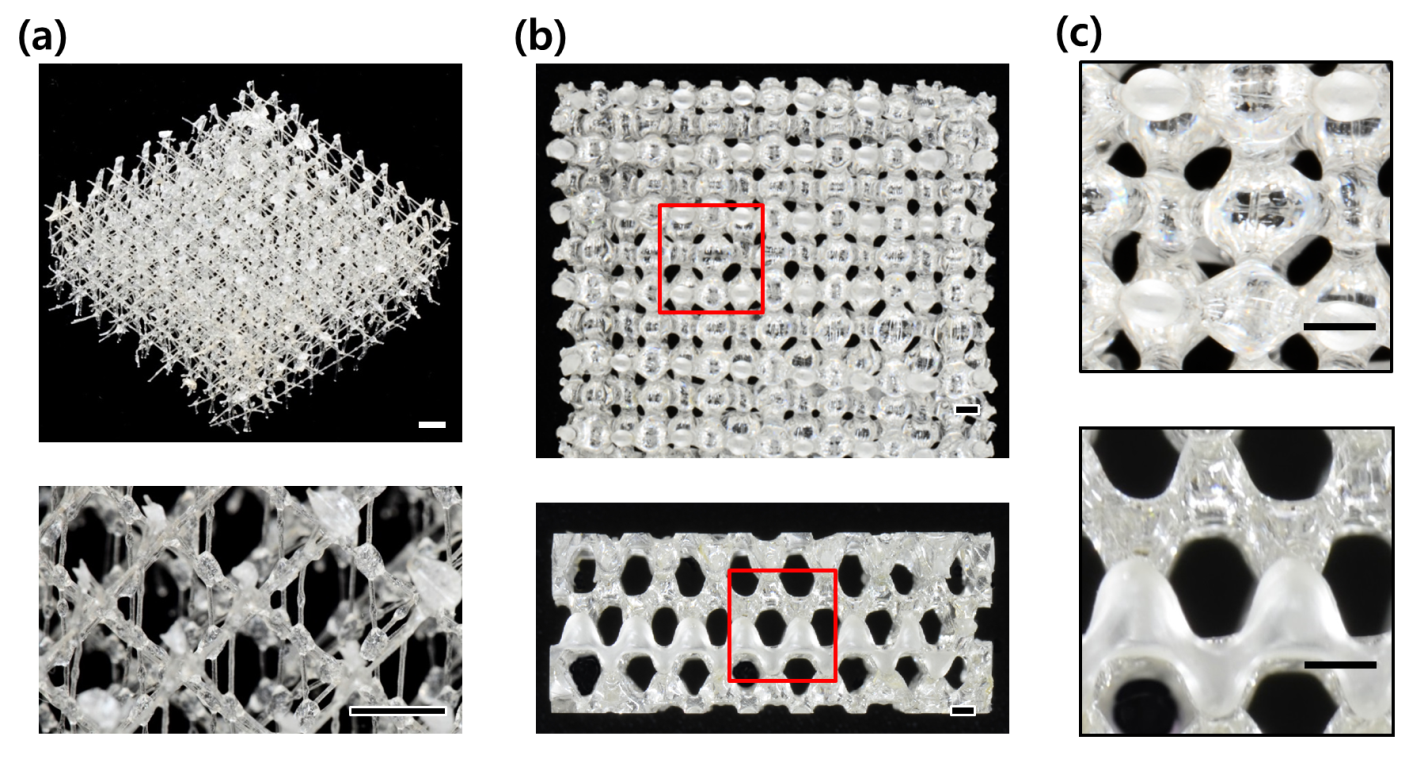


Figure S10. Template formation for the *D*-surfaced Shellular specimens. (a) A wire-woven Kagome and its close-up with the intersections fixed by infiltrating a UV curable resin. (b) and (c) Top/side views and their close-ups of a completed template. Scale bars, 3*mm*
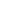
(a), 2*mm* (b, c).

**Movie S1**

2D schematic showing how the weaving process operates.

**Movie S2**

A movie illustrating the 3D configuration of the specimen measured by micro-CT.

**Movie S3**

In-situ compression video (played at 10x speed) of a Shellular specimen at the relative density of *ρ/ρ_s_* = 2.6* 10^-4^ (cell size: 3mm; wall thickness: 0.3μm) to ~25% strain. It demonstrates the stable and fully recoverable behavior.

**Movie S4**

In-situ compression video (played at 10x speed) of a Shellular specimen at the relative density of *ρ/ρ_s_* = 1.2* 10^-2^ (cell size: 3mm; wall thickness: 16.4μm) to ~25% strain. It shows the brittle fracture of the shell under compression.
