# Supplementary figures and images for: A Microscopic Shell Structure with Schwarz’s D-Surface
Source: Sci Rep. 2017 Oct 17;7:13405. doi: 10.1038/s41598-017-13618-3 (PMC5645438; doi:10.1038/s41598-017-13618-3)

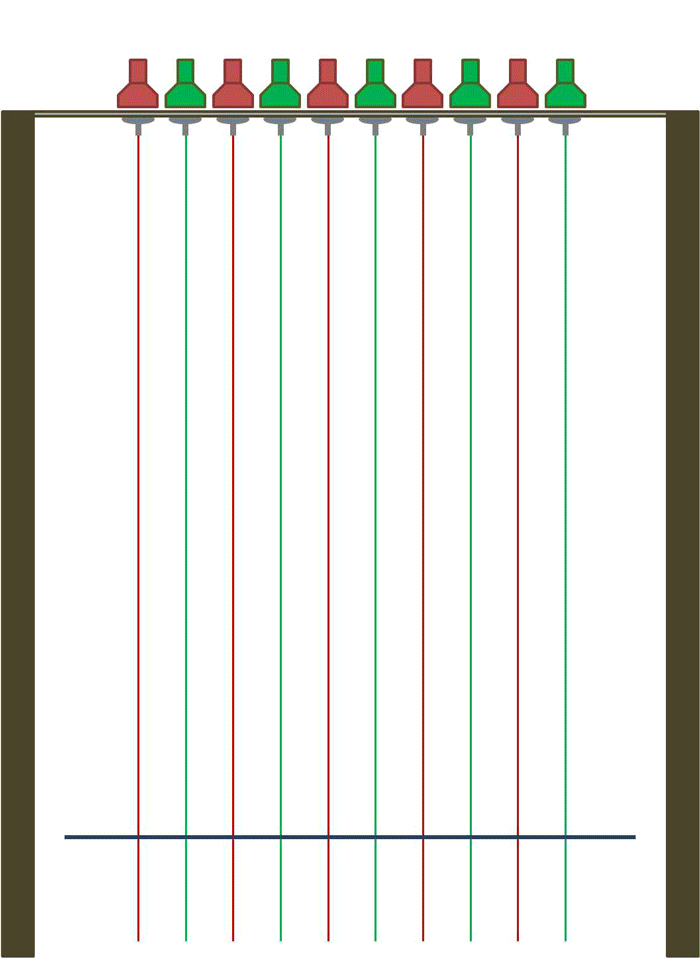

Supplement: Supplementary file 2 — Movie S1 [file 41598_2017_13618_MOESM2_ESM.gif]
